# The Effect of Epidural Analgesia on Quality of Recovery (QoR) after Open Radical Nephrectomy: Randomized, Prospective, and Controlled Trial

**DOI:** 10.3390/jpm14020190

**Published:** 2024-02-08

**Authors:** Ruben Kovač, Ivo Juginović, Nikola Delić, Ivan Velat, Hrvoje Vučemilović, Ivan Vuković, Verica Kozomara, Angela Lekić, Božidar Duplančić

**Affiliations:** 1 Department of Anesthesiology and Intensive Care, University Hospital Split, 21000 Split, Croatia; ndelic@kbsplit.hr (N.D.); hvucemilovic@kbsplit.hr (H.V.); ivukovic@kbsplit.hr (I.V.); vkozomara@kbsplit.hr (V.K.); 2 Department of Urology, University Hospital Split, 21000 Split, Croatia; ijuginovic@kbsplit.hr (I.J.); ivelat@kbsplit.hr (I.V.); 3 Surgery Department, University Hospital Split, 21000 Split, Croatia; apalavra@kbsplit.hr

**Keywords:** quality of recovery, radical nephrectomy, general anesthesia, epidural anesthesia, postoperative analgesia, morphine, ropivacaine, tramadol, multimodal analgesia

## Abstract

No studies are currently evaluating the quality of recovery (QoR) after open radical nephrectomy (ORN) and epidural morphine analgesia. This was a randomized, prospective, and controlled study that explored the QoR on the first postoperative day after ORN. Eighty subjects were randomized into two groups. The first group received general anesthesia combined with epidural anesthesia and postoperative epidural analgesia with morphine and ropivacaine. The second group received general anesthesia and continuous postoperative intravenous analgesia with tramadol. Both groups received multimodal analgesia with metamizole. The primary outcome measure was the total QoR-40 score. The secondary outcome measures were QoR-15, QoR-VAS, and the visual analog scale (VAS) for pain, anxiety, and nausea. The median difference in the QoR-40 score after 24 postoperative hours between the two groups of patients was 10 (95% CI: 15 to 5), *p* < 0.0001. The median score and IQR of QoR-40 during the first 24 postoperative hours in the epidural group was 180 (9.5), and in the control group, it was 170 (13). The general independence test for secondary outcomes between groups was significant (*p* < 0.01). QoR-VAS was correlated with QoR-40 (r = 0.63, *p* ≤ 0.001) and with QoR-15 (r = 0.54, *p* ≤ 0.001). The total QoR-40 and QoR-15 alpha coefficients with a 95% CI were 0.88 (0.85–0.92) and 0.73 (0.64–0.81), respectively. There was a significant difference in the QoR between the epidural and the control groups after ORN. The QoR-40 and QoR-15 showed good convergent validity and reliability.

## 1. Introduction

The postoperative quality of recovery (QoR) is an important aspect of postoperative care after radical nephrectomy. Renal cell carcinoma (RCC) is the most common type of kidney cancer, accounting for about 85% of all cases. It is a highly aggressive cancer that is often resistant to chemotherapy and radiotherapy [1]. Radical nephrectomy is important in the management of the renal neoplasm. Tumor diagnosis is incidental in 60% of cases [2,3]. Some 36% of cases at stage III or IV at the time of diagnosis are without symptoms. As many as 22% of kidney cancers at the time of diagnosis are metastatic [3]. The 5-year survival rate drops from 93% to 12% when the cancer has spread to distant parts of the body outside of the kidney [4].

Postoperative analgesia after open nephrectomy can be administered with intrathecal morphine [5], thoracic epidural analgesia [5,6], continuous wound infiltration [6], and paravertebral analgesia [7]. Regional anesthesia with an ultrasound-guided block is also an alternative in postoperative pain therapy, in the form of an erector spinae plane block at the T10 transverse process level [8] and a subcostal anterior quadratus lumborum block [9].

Regional anesthesia under ultrasound guidance is also attractive for pain management in laparoscopic nephrectomy using an anterior quadratus lumborum block at L2 level [10] and a lateral quadratus lumborum block [11].

This study explores the effects of two types of multimodal analgesia after open radical nephrectomy. Metamizol (dipyrone) was used as a part of multimodal analgesia in both groups. Metamizol has analgesic, antipyretic, and spasmolytic properties [12].

The first group in our study evaluated combined general anesthesia with epidural ropivacaine anesthesia followed by morphine epidural analgesia. Although epidural morphine is well known and clinically utilized for analgesia following a cesarean section [13,14,15], there is no research concerning epidural morphine analgesia after ORN on quality of recovery-40 (QoR-40). Morphine is a hydrophilic opioid that, given in epidural form, results in prolonged analgesia—up to 24 h [16]. Our previous work [17] evaluated epidural morphine analgesia and postoperative QoR-40 after radical prostatectomy.

There are no studies regarding QoR-40 and multimodal analgesia with intravenous tramadol and metamizole after ORN. Tramadol active metabolite (0-desmethyltramadol) acts as a weak mu-opioid agonist [18]. Tramadol (+) enantiomer inhibits serotonin reuptake, and (−) enantiomer inhibits norepinephrine reuptake. There is analgesic synergy between monoaminergic modulation and opioid agonism. Unlike strong opioids, tramadol has minimal depressive effect on respiratory function, especially after laparoscopy or thoracotomy, and low incidence of constipation. Tramadol, unlike morphine, does not depend on kidney function. Tramadol analgesia is partly antagonized by naloxone. A selective 5HT5 receptor antagonist, ondansetron, reduces the analgesic effect. The analgesic effect of tramadol can increase when it is combined with drugs such as paracetamol, metamizole, or ketorolac [19]. Tramadol’s analgesic potency is about 10% of that of morphine [18,19], but the analgesic potency depends on the patient’s CYP2D6 function. An increased CYP2D6 function increases analgesia and the risk of toxicity, while a lower function reduces analgesia [20].

Postoperative QoR can be evaluated using psychometric questionnaires such as the QoR-40 scale and the QoR-15. The QoR-40 measures patients’ health status after surgery and anesthesia, and it has been proposed as a measure of outcome in clinical trials [21]. The QoR-40 questionnaire is multidimensional: it includes emotional state (eight items), physical comfort (twelve items), physical independence (five items), psychological support (seven items), and pain (seven items). The QoR-40 scale is widely utilized in numerous clinical trials and has received extensive validation [22,23,24,25,26,27].

The primary objective of this study was to assess two anesthetic procedures, namely, general anesthesia followed by IV analgesia, and general and epidural anesthesia followed by epidural analgesia, with reference to the postoperative QoR-40 for patients undergoing radical nephrectomy. The secondary goal was to assess QoR using secondary outcomes: the QoR-40 dimensions score, QoR-15, VAS-QoR, and visual analog scales (VAS) for nausea, anxiety, and pain.

## 2. Materials and Methods

This was a single-centered, randomized, prospective, and controlled clinical trial. Examiners were blinded in the postoperative period. The study was approved by the ethics committee of the University Hospital of Split, and the trial was registered under the number NCT04521556. In this study, participating patients were scheduled for elective open radical nephrectomy between April 2019 and April 2021.

Subjects were excluded if they had absolute contraindications for epidural anesthesia, dementia, a Montreal Cognitive Test score of less than 24, or intraoperative complications requiring postoperative intensive care unit admission, or if they declined to take part in the study. Subjects were informed about the study one day before surgery and their informed consent was sought. Patients were randomized to receive epidural or control treatment using the R program Blockarand, which created randomization cards [28]. The randomization cards for treatment assignments were printed out and sealed in envelopes. The envelopes were designated with an ordinal number on their exterior, but the randomization cards inside them were not visible. Closed envelopes were opened by an examiner after the enrollment process and before the administration of the anesthesia.

All participants received diazepam 5 mg p.o. 12 h and 1 h before the surgery to decrease preoperative anxiety and increase comfort. 

Low-weight heparin (4000–6000 IU), depending on body weight, was given 12 h before the surgery. The subjects and infusions were warmed to avoid hypothermia. The general anesthesia for tracheal intubation was induced with midazolam 2.5 mg, fentanyl 100 μg, propofol 1–2 mg/kg, and vecuronium 0.1 mg/kg. Hypovolemia was treated using balanced crystalloids. In exceptional cases, 6% hydroxyethyl starch was used before administering blood transfusion products to treat profound hypovolemia. Blood transfusions were given in cases of blood loss or other clinical indications. Bradycardia was treated with atropine. Hypotension related to anesthesia was treated with ephedrine boluses. Reversion of the muscular blockade was achieved by administering neostigmine 2.5 mg and atropine 1 mg.

Participants were placed in a urology high-care unit for one day and were provided with non-invasive monitoring and continued nursing care. Crystalloid infusions were used to maintain diuresis. Pantoprazole 40 mg was given for gastroprotection, and metoclopramide 10 mg was given for the prevention of nausea and vomiting.

The epidural group received general anesthesia combined with epidural anesthesia.

Epidural catheter insertion was performed before the induction of general anesthesia using a midline approach at Th 11–Th 12 level or Th 10–Th 11 level. A safety check on the inserted epidural catheter to confirm the exclusion of intradural anesthesia was performed using lidocaine 60 mg. The practical advantage of an epidural catheter compared to single shut epidural anesthesia is the facility for the subsequent addition of local anesthetic to achieve the desired level of epidural anesthesia. Gradual local anesthetic titration prevents an extensive neural blockade. Later, if surgery is prolonged, adding more local anesthetic prevents block regression.

Spinal morphine is an alternative to postoperative analgesia, but with the slow onset of morphine analgesia, it provides no intraoperative analgesia.

The epidural anesthesia mixture consisted of ropivacaine 6.5 mg/mL and fentanyl 8.3 μg/mL. Ropivacaine was the principal epidural drug. Epidural loading dosages of 3 or 4 mL were carefully given to ASA III and elderly patients; 5 mL was given to ASA II and 6 mL to ASA I and young patients, and the dosage was titrated afterwards using the epidural boluses (1–2 mL).

After the induction of the general anesthesia, the general anesthesia was performed with isoflurane in a mixture of 50/50 nitrous oxide and oxygen to achieve a minimum alveolar concentration between 0.6 and 0.8. 

Before the end of the anesthesia, participants received 3.2 mg of morphine epidural analgesia. The epidural mixture was composed of ropivacaine 4.4 mg/mL and morphine 0.8 mg/mL. Further postoperative epidural analgesia was given in boluses for the following 24 h as a mixture of morphine 0.4 mg/mL and ropivacaine 2.2 mg/mL. The principal drug for epidural analgesia was morphine. The ropivacaine dose was very low. Epidural analgesia was administered by a urologist in accordance with our classification based on morphine dosage: reduced dosage—class I (2 × 0.8 mg), intermediate dosage—class II (2 × 1.2 mg), and high dosage—Class III (3 × 1.2 mg). The reduced dosage was given to ASA III and elderly patients, the intermediate dosage to ASA II, and the high dosage to ASA I or young patients.

The control group included patients who received balanced general anesthesia followed by postoperative intravenous tramadol analgesia.

The balanced general anesthesia was performed with nitrous oxide and oxygen in a 50/50 mixture and isoflurane to achieve the minimum alveolar concentration between 0.8 and 1. The loading dose of fentanyl was between 6 and 8 mcg/kg. If necessary, additional fentanyl doses were administered incrementally, as needed. The dosage of postoperative IV tramadol analgesia was 100 mg in the first hour followed by 300 mg continuously for the next 24 h. To achieve multimodal analgesia, metamizole 2.5 g was administered intravenously before the end of the surgery and 12 h later in both groups.

The surgery was performed in the classical flank position. A flank incision above the 11th or 12th rib was made with dissection of the flank musculature (latissimus dorsi, serratus posterior inferior, external and internal oblique muscles) to expose the 11th or 12th rib, after which the intercostal muscles were incised prior to the rib resection. The peritoneal fold was reflected medially and the transverse muscle was divided to expose the retroperitoneal area and Gerota’s fascia. Furthermore, the kidney was mobilized, and the renal artery, renal vein, and ureter were ligated and cut. The kidney was removed, and the wound was closed with a drain set through a separate stab wound well below the 12th rib.

The primary outcome in this study is quality of recovery-40 (QoR-40) total score. The total score represents the global quality of recovery. The minimum possible score is 40, and the maximum possible score is 200. The QoR-40 is a questionnaire for patients about their recovery in the last 24 h. The QoR-40 has five sections (see Table 1). Section scores were used as secondary outcomes. The pain section has two items regarding moderate and severe pain that are in the context of surgery, but the other five items have a more general context related to postoperative care.

The minimum clinically important difference (MCID) is the smallest change in an outcome that is considered relevant by patients [29]. If the difference in QoR-40 score is less than the MCID value of 6.3 points, then the effect size is not important, regardless of statistical significance. The MCID is useful in data interpretation because it differentiates statistical difference from clinical difference. Regarding data variability, previous work has shown the mean and standard deviation of the QoR-40 score in a major surgery group to be 166 ± 15 [30]. Our educated guess about sample size calculation, effect size, and data variability was made following a study by Catro-Alves et al. [31]. We computed that a sample size of 62 was necessary for a 10 point mean difference in the QoR-40 score to achieve an alpha of 0.05 and a power of 0.8.

In this study, the total QoR-15 score, QoR-VAS, and VAS for pain at rest, pain on activity, and anxiety are used as secondary outcomes.

The QoR-15 is a shorter version of the QoR-40 questionnaire, consisting of 15 items scaled from 0 to 10. The minimum score is 0, and the maximum is 150. Shorter and easier to fill out, it consumes less time than the QoR-40 [32]. The QoR-15 has been used in recent clinical trials [33,34,35]. 

The QoR-VAS was used for a validation study for the QoR-40 and QoR-15. It is a patient-rating visual analog scale. Recovery is quantified by placing an “X” on the line. The range of the line representing the score is from 0 to 100 mm. Poor recovery is represented on the left-hand side and is defined as severe pain, nausea and vomiting, confusion, immobilization, eating difficulties, and problems with communication. Excellent recovery is represented on the right-hand side and is defined as no pain, comfortable, alert, active, enjoying food, and communicating freely.

Visual analogue scales for pain at rest, pain on activity, and anxiety were evaluated 24 h after the surgery. The patient’s acceptable symptom state for VAS pain at rest is 33 points or less [36]. We consider a score of VAS pain on activity of less than 40 points to be acceptable. A VAS anxiety greater than 34 indicates an anxious patient [37].

A VAS for nausea intensity estimated the worst nausea intensity over 24 h. We consider a VAS nausea score of less than 30 points to be acceptable.

The authors translated the QoR-40 and QoR-15 from English to Croatian. A native English speaker translated them back. The simple content of the QoR instruments makes changes in semantics unlikely [38,39]. We tested the reliability and convergent validity of our Croatian versions of the QoR-40 and QoR-15. The correlation between the QoR-VAS and the QoR-40 and QoR-15 was considered to represent the convergent validity of the instruments. The reliability of the QoR-15 and QoR-40 was tested using the Cronbach α test for internal consistency.

All data were analyzed with descriptive statistics. The normality of the data was tested using the Mardia test [40], the univariate Shapiro–Wilk test, and QQ plots. Fisher’s exact test was used for categorical variables. Normal data are presented as mean ± standard deviation. Abnormally distributed data are displayed as the median (interquartile range). Boxplot was used for data visualization. Abnormally distributed data were tested using the Wilcoxon rank test and Spearman’s rank correlation test. The approximate general independence test was used for nonparametric multivariate analyses [41]. A separate general independence test was used for secondary QoR outcomes and for secondary VAS outcomes. The purpose of this robust test is to decrease Type I errors. A *p*-value less than 0.05 is considered statistically significant. A further analysis of QoR-40 dimensions and secondary outcomes was performed using the Wilcoxon rank test, and *p* values were corrected with the Holm test. To facilitate the interpretation of effect size, statistical estimates were presented as differences in medians and Spearman r effect sites. A statistical analysis was performed using the R program with RStudio. The R code is provided in Supplementary Materials.

## 3. Results

### 3.1. Participant Flow

**Figure 1:**
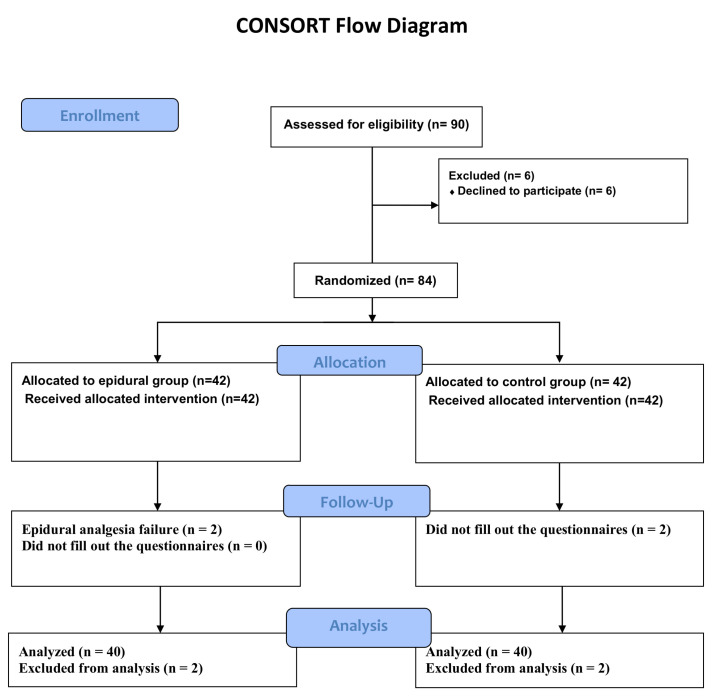
Flow diagram of the participants of the study.

Between April 2019 and April 2021, 80 out of the 90 subjects originally randomized during that period completed the study (Figure 1). Two of the patients in the control group refused to fill out questionnaires. In the epidural group, two patients failed epidural analgesia.

### 3.2. Descriptive Statistics

The mean age of patients was 67 ± 14.3 years. The mean surgery time was 133 ± 26 min. The QoR-40 median score was 175.5 (165.8–181.0).

The QoR-15 and QoR-VAS median scores were 114.5 (104–112) and 78.5 (72–82), respectively. The median score of the VAS pain during rest was 20 (0–20). The median score of the VAS pain during activity was 40 (30–50). The median scores of VAS anxiety and VAS nausea were 0 (0–20) and 0 (0–20), respectively. The VAS nausea score in our study was above 30 points in 21.25% of the patients. Baseline data between the groups is presented in Table 2.

### 3.3. Primary Outcome

**Figure 2:**
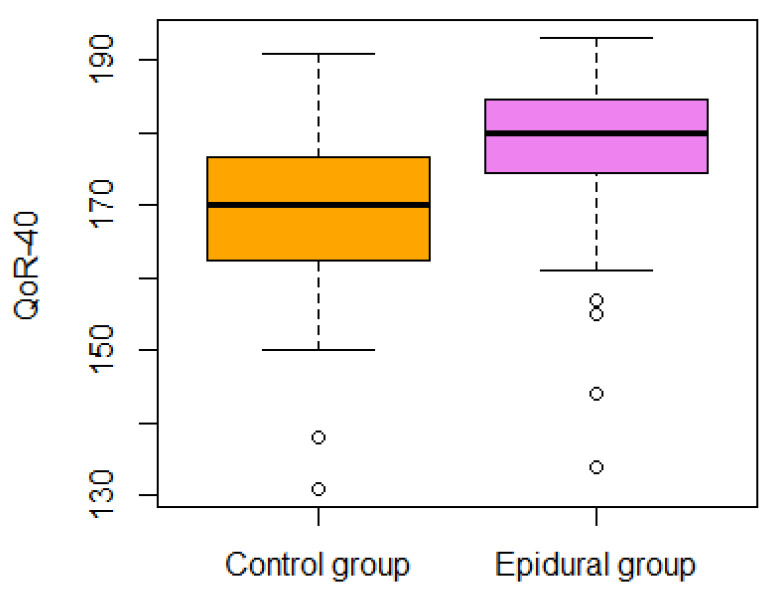
Global QoR-40 scores in the epidural and control group.

The median difference in the global QoR-40 score during the first 24 postoperative hours between the epidural group and the control group was 10 (95% CI: 15 to 5), *p* < 0.0001. The median score and IQR of the QoR-40 during the first 24 postoperative hours in the epidural group was 180 (9.5), and in the control group, it was 170 (13) (see Figure 2). The Spearman’s r was 0.43. The QoR-40 dimensions score are presented in Table 3.

**Figure 3:**
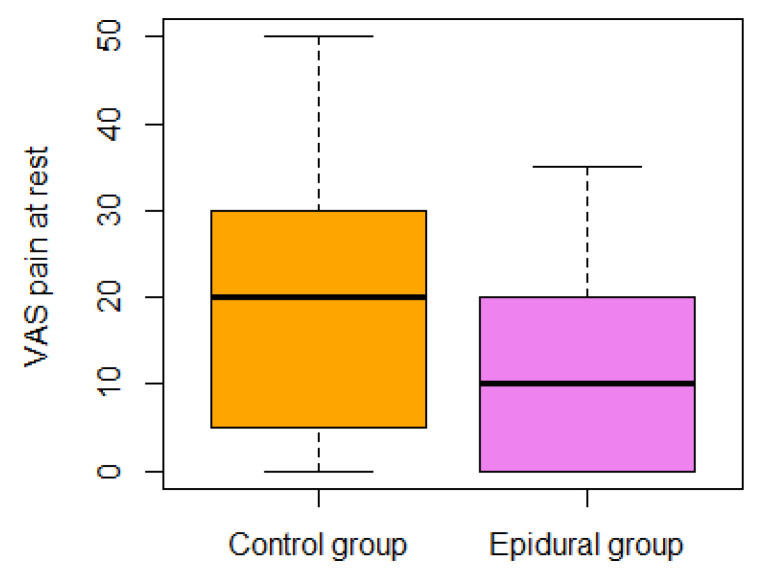
The VAS pain at rest score was examined at the end of the analgesia protocol.

**Figure 4:**
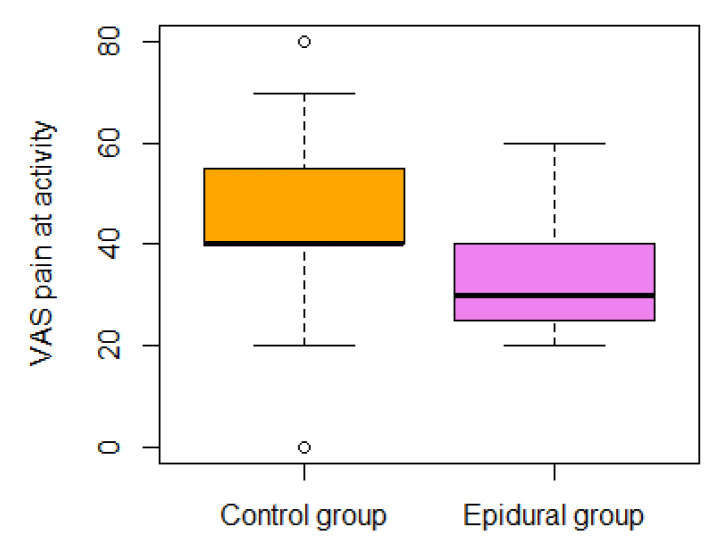
The VAS pain on activity was examined at the end of the analgesia protocol.

**Figure 5:**
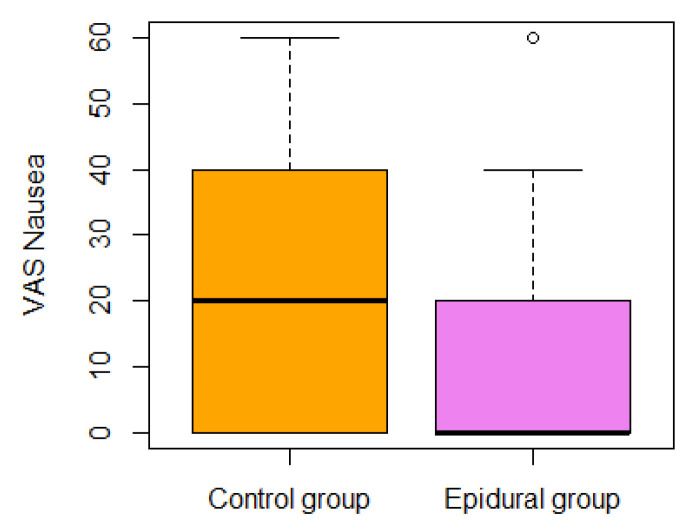
The VAS nausea intensity measured the worst patient score on the first postoperative day.

The general independence test for secondary QoR outcomes was significant (*p* < 0.001). A further analysis of secondary outcomes is presented in Table 4. The general independence test for VAS outcomes (pain on activity, pain at rest, anxiety, and worst nausea in 24 h) was significant (*p* = 0.0008) (see Figure 2, Figure 3, Figure 4 and Figure 5). In the epidural group, there was nausea in 30% vs. 55% in the control group. 

The VAS pain at rest score was significantly lower than 30 points in the one-sided Wilcoxon rank test, *p*-value < 0.001. VAS pain in the activity score was not significantly lower than 40 points in the one-sided Wilcoxon rank test, *p*-value = 0.3.

The QoR-VAS was strongly correlated with the QoR-40 (r = 0.63, *p* ≤ 0.001) and with the QoR-15 (r = 0.54, *p* ≤ 0.001). A negative correlation was found between secondary outcomes. Correlations are presented in Table 5.

The QoR-40 and QoR-15 alpha coefficients with a 95% CI were 0.88 (0.85–0.92) and 0.73 (0.64–0.73), respectively. Both alpha coefficients were above the recommended value of 0.7.

## 4. Discussion

This study showed that the epidural group achieved a higher QoR-40 score than the control group.

The median score difference was higher than an MCID value of 6.3 points, and the score difference was meaningful. A further analysis of the QoR-40 dimensions has shown that the epidural group had higher scores for physical comfort and emotional state. The psychological support and physical independence scores were similar in both groups. Psychological support depends on nurses, doctors, and the patient’s family. The physical independence score is affected by extensive surgery as subjects are mostly undergoing bed rest. Pain score differences between groups were small and pain score distribution tended towards maximum scores. 

The secondary outcomes, the QoR-15, QoR-VAS, VAS pain at rest and VAS pain on activity, did differ between the groups. The QoR-15 median score difference was more than the MCID value of 8. The VAS pain scores after 24 h in both groups were acceptable at rest (<30). The VAS pain scores on activity were higher than our expected cutoff of 40 points. It is not clear what is optimal VAS pain in strain and activity, but less pain in activity is better for early recovery. Both VAS measures were taken 24 h after surgery, representing just one time point in the QoR evaluation. The VAS scale is unidimensional; it does not cover affective and cognitive aspects of pain. Pain can evoke strong affective responses, including fear, anxiety, depression, anger, and frustration, with an impact on a person’s overall well-being. The cognitive dimension involves the thoughts, beliefs, and attitudes that influence how a person perceives and copes with pain. There are also behavioral and sociocultural dimensions [42]. 

The VAS anxiety and VAS nausea difference scores between groups were small. The worst VAS nausea score was evaluated in a retrospective way. The VAS nausea score revealed a high incidence of nausea in the tramadol group. Although classical analgesia studies only explore pain intensity scores, nausea can also decrease the QoR-40 score.

The secondary outcome correlations supported the convergent validity of the QoR-40 and QoR-15. We have decided to use the QoR-40 on the first operative day as the primary outcome because it is simple and comprehensive. Filling out the questionnaire does not cause patients concern, because it is presented only once and the questions are retrospective. 

Partial nephrectomy is the gold-standard treatment for T1a–b tumors where it is technically feasible to save kidney function. Despite its minimally invasive techniques, open partial nephrectomy is an important surgical skill for a smaller proportion of renal masses in the setting of more complex renal tumors [43]. 

While laparoscopic and robot-assisted nephrectomies are preferred surgical approaches because of the shorter hospital stay and the lower need for blood transfusion compared to open nephrectomy, there is no oncological difference in either of these approaches [44]. If minimally invasive surgery may compromise oncological, functional, or perioperative outcomes, then open surgery is advocated [45].

Open radical nephrectomy (ORN) is reserved for larger and more complex tumors [46]. Cytoreductive nephrectomy is reserved as an option for metastatic renal cell carcinoma [46]. Open nephrectomy (ON) is still performed in a quarter of cases [47].

The postoperative pain scores and incidence of chronic postsurgical pain are similar in both open nephrectomy and laparoscopic nephrectomy [48].

Different surgical techniques have different effects on the QoR-40 on the first postoperative day. Our randomized control study explored the effect of analgesia with morphine epidural analgesia versus tramadol analgesia after open radical prostatectomy. There was no difference in the QoR-40 score between groups. The median QoR-40 score for both groups was 181 (177–188), and the median VAS pain on activity score was 40 (20–40) [17]. A retrospective study by Kobari et al. evaluated the QoR-40 after robot-assisted partial nephrectomy using a retroperitoneal or transperitoneal approach. The retroperitoneal approach achieved a higher score (163.4 ± 23.7) than the transperitoneal approach (156.2 ± 23.7) on the first postoperative day [49].

The QoR is an important outcome for encouraging living kidney donation. A randomized control study by Bruintjes et al. tested the effect of intraoperative deep vs. moderate muscle relaxation during laparoscopic donor nephrectomy. The QoR-40 score on the first postoperative day was not statistically significant (169 ± 18 vs. 169 ± 15). The deep relaxation group had lower pain scores at 6 h, 24 h, and 48 h after surgery, with a similar analgesia requirement [50].

Han et al. investigated propofol-based TIVA and sevoflurane anesthesia after laparoscopic donor nephrectomy. Both groups received intrathecal morphine (0.2 mg) and postoperative PCA (fentanyl and ramosetron) infusions. The propofol group had a better QoR-40 score of 169 (162–179) vs. 142 (131–154). Nausea and vomiting were noted in 30% of subjects in the propofol group and 65% in the sevoflurane group [51].

Yoon et al. explored the effects of propofol-based TIVA and desflurane anesthesia in laparoscopic nephrectomy. The TIVA group had a better QoR-15 score than the desflurane group on the first and second days but not on the third postoperative day [52].

One of the limitations of our study is the fixed dosage of tramadol, which may have negative implications for QoR. Depending on the CYP2D6 activities, some patients have a higher risk of tramadol side effects, and some experience a decreased analgesic effect. 

We excluded patients older than 80 years who were more sensitive to tramadol and morphine. Despite the cumulative morphine dose in this study being low, we excluded subjects with poor glomerular filtration.

Two patients had failure of epidural analgesia and did not finish the study, but the initial study protocol was not designed to deal with an analysis of the intention to treat. Epidural catheters, like all other catheters used for regional anesthesia, could be malpositioned, with resulting analgesia failure. 

Both tramadol and morphine for epidural analgesia can lead to postoperative nausea and vomiting. The postoperative PONV prophylaxis was less than is recommended [37]. At least two antiemetic drugs are recommended for the prevention of nausea and vomiting. More aggressive antiemetic therapy is necessary for a better QoR. Propofol-based TIVA for general anesthesia could improve the QoR and decrease PONV.

We did not use paracetamol for multimodal analgesia. Paracetamol could improve tramadol and metamizole analgesia.

We did not blind examiners to group allocation nor patients to group allocation, but we made sure that authors who collected questionnaires did not perform anesthesia or postoperative analgesia.

This was a patient-centered study that should further inform clinical practice about two different approaches in open radical nephrectomy. This is the first study about morphine-based epidural analgesia after open radical nephrectomy as multimodal analgesia. There was a significant difference in the QoR-40 score between the groups, with the morphine epidural group achieving higher QoR-40 scores. The morphine epidural group had a good QoR-40. Epidural morphine analgesia does not need an infusion pump and is simpler than continuous epidural analgesia. Although the tramadol group achieved lower QoR-40 scores, tramadol analgesia is easy to perform and can be an alternative if epidural anesthesia is not feasible.

More studies regarding the quality of recovery of patients after open radical nephrectomy are necessary.

## Figures and Tables

**Table 1:** Quality of recovery-40: five sections and items.

Quality of Recovery-40
**Emotional state (9 items)**
Feeling comfortable
Having a general feeling of well-being
Feeling in control
Bad dreams
Feeling anxious
Feeling angry
Feeling depressed
Feeling alone
Difficulty falling asleep
**Physical comfort (12 items)**
Able to breathe easily
Have had a good sleep
Being able to enjoy food
Feeling rested
Nausea
Vomiting
Dry retching
Feeling restless
Shaking or twitching
Shivering
Feeling too cold
Feeling dizzy
**Psychological support (7 items)**
Able to communicate with hospital staff
Able to communicate with family or friends
Getting support from hospital doctors
Getting support from hospital nurses
Having support from family or friends
Able to understand instructions or advice
Feeling confused
**Physical independence (5 items)**
Able to return to work or usual home activities
Able to write
Have normal speech
Able to wash, brush teeth, or shave
Able to look after own appearance
**Pain (7 items)**
Moderate pain
Severe pain
Headache
Muscle pains
Backache
Sore throat
Sore mouth

**Table 2:** Baseline data between the groups. Data are presented as mean ± SD, n (%), or median (interquartile range). Data were compared between groups using the independent *t* test, the Mann–Whitney U test, or Fisher’s exact test. * Includes time for placing epidural catheter.

Baseline Characteristic and Intraoperative Data			
	Epidural Group	Control Group	*p*-Value
	n = 40	n = 40	
Age (years)	70.5 ± 13.25	66.6 ± 12.25	0.1
Male	28	26	0.63
Female	12	14	
ASA physical status (n)			
I	2 (5%)	4 (10%)	
II	26 (65%)	24(60%)	0.76
III	12 (30%)	12(30%)	
Surgery duration (minutes)	130 ± 30	130 ± 30	0.48
Anesthesia duration (minutes)	155 * ± 40	160 ± 40	0.88
Red Blood Cells			
0	39 (97.5%)	38 (95%)	1
1 unit	0 (0%)	1(2.5%)	
2 units	1 (2.5%)	1 (2.5%)	
Crystalloids (mL)	1600 (250)	1700 (300)	0.15
Vecuronium (mg)	12 (2)	14 (3.25)	<0.001
Fentanyl (mcg)	100 (0)	500 (200)	
Epidural analgesia	Class I (50%)		
	Class II (37.5%)		
	Class III (12.5%)		

**Table 3:** QoR-40 dimensions. Data are presented as medians (IQR). The Wilcoxon rank sum test was used for the statistical estimate. Holm correction was used for *p* values corrected for multiple tests.

QoR-40 Dimensions Score					
	Epidural Group	Control Group	Statistic Estimate		
Dimensions	n = 40	n = 40	Difference in Medians	Effect Size	*p* Value
Physical comfort	52(4)	49(6.5)	−4	r = 0.43	0.0001 *
Emotional state	42 (5.3)	38.5 (8)	−3	r = 0.31	0.005 *
Pain	33.5(2)	33 (1)	−1	r = 0.26	0.018
Psychological support	35 (1)	34.5 (2)	0	r = 0.09	0.43
Physical independence	18 (4.5)	17 (3.3)	−1	r = 0.18	0.12

* Statistical significance with Holm correction.

**Table 4:** Secondary outcomes. Data are presented as medians (IQR). The Wilcoxon rank sum test was used for the statistical estimate. Holm correction was used for *p* values corrected for multiple tests.

Secondary Outcomes					
	Epidural Group	Control Group	Statistic Estimate		
	n = 40	n = 40	Difference in Medians	Effect Size	*p* Value
QoR-15	119 (10.5)	106 (18)	−13	r = 0.49	<0.008 *
QoR-VAS	81.5(14.25)	75 (8)	−4	r = 0.26	0.022
VAS pain at rest	10 (20)	20 (22.5)	10	r = 0.32	<0.0125 *
VAS pain on activity	30 (12.5)	40 (12.5)	10	r = 0.43	<0.01 *
VAS nausea	0 (20)	20 (40)	3 × 10^−5^	r = 0.27	0.018
VAS anxiety	0 (0)	0 (20)	3 × 10^−5^	r = 0.23	0.04

* Statistical significance with Holm correction.

**Table 5:** Correlational analysis of secondary outcomes.

VAS	Nausea	Pain at Rest	Pain on Activity	Anxiety
QoR-40	−0.56 **	−0.33 **	−0.30 **	−0.42 **
QoR-15	−0.56 **	−0.34 **	−0.34 **	−0.34 **
QoR-VAS	−0.29 **	−0.32 **	−0.25 *	−0.25 *

** *p* ≤ 0.01. * *p* < 0.05.

## Data Availability

The data presented in this study are available on request from the corresponding author. The data are not publicly available due to ethical restrictions.

## Supplementary Materials

The following supporting information can be downloaded at: https://www.mdpi.com/article/10.3390/jpm14020190/s1.
