# Supplementary material for: The Effect of Epidural Analgesia on Quality of Recovery (QoR) after Open Radical Nephrectomy: Randomized, Prospective, and Controlled Trial
Source: J Pers Med. 2024 Feb 8;14(2):190. doi: 10.3390/jpm14020190 (PMC10890626; doi:10.3390/jpm14020190)
Supplement: Supplementary file 1 [file jpm-14-00190-s001.zip › jpm-2843327-supplementary.pdf]

R code copy pasted from R script

#Not all code here provided was used in data analysis but it can be used in similar research.

#R packages that need to be installed and loaded

```
citation("npmv") # for citation
library(npmv)
library(tidyr) #for short to long data form
library("MPsychoR")
library("psych")
library("reshape2")
library(sjPlot)
library(PerformanceAnalytics)
library(corrplot)
library(MVN)
library(lavaan)
library(semPlot)
library(semTools)
library(tidyverse)
library(ggpubr)
library(rstatix)
library(DescTools)
library(coin)
library(ggplot2)
library(mvnormtest)
library(pwr)
library(effectsize)
```

#import data in R environment in a TXT file.

I have to copy and paste all the variables from "Excel" - without column name, just #numbers. R studio is automatically naming it with letter V and ordinal numbers: V1,V2,V3...

It is important to know the names for renaming variables in R studio

R studio - import dataset- from Text ...

Dealing with QoR-40 questioner:

"Natural" order of items and dimensions from QoR-40 survey

#Emotional state: 2,9,12,28,36,37,38,39,40

#Physical comfort: 1,5,10,11,19,20,21,24,25,26,27,34

```
#Psychological support: 13,14,15,16,17,18,35

#Physical independence: 3,4,6,7,8

#Pain: 22,23,29,30,31,32,33

# in our excel items are rearranged to follow the dimensions, not the natural order from QoR-40 questioner

# To create dimensions sum score we need to sum score of the items.

bubrezi.vizualizacija <- read.delim("C:/Users/38591/OneDrive/Desktop/bubrezi vizualizacija.txt", header=FALSE)   #import txt file


q= bubrezi.vizualizacija

#make summary dimensional score

q$Comfort = as.numeric(apply(q[,1:12], 1, sum))

q$Emotions = as.numeric(apply(q[,13:21], 1, sum))

q$Independence= as.numeric(apply(q[,22:26], 1, sum))

q$PschyologicalSupport= as.numeric(apply(q[,27:33], 1, sum))

q$Pain= as.numeric(apply(q[,34:40], 1, sum))


#The sum of QoR score = global QoR score

# Creating the global score sum and name. First 40 columns are QoR-40 items, next 15 are QoR-15 items

q$QoR40= as.numeric(apply(q[,1:40], 1, sum))

q$QoR15= as.numeric(apply(q[,41:55], 1, sum))

#naming other variables

        q$QoRVAS <- q$V56

        q$Nausea <- q$V57

        q$PainR<- q$V58

        q$PainA<- q$V59

        q$Anxiety<- q$V60

        #q$Epidural <- factor(q$V61 )

        q$Epidural[q$V61 == '0'] <- 'Control  group' # changing name of binary variable outcome

        q$Epidural[q$V61 == '1'] <- 'Epidural group'

        q$subject<- factor(q$V62)
```

```
q$Age<- q$V63
q$ASA<- as.integer(q$V64) # ASA is ordinal variable.
q$Timeanest<- as.integer(q$V65)
q$Timesurgery<- as.integer(q$V66)
q$fentanyl<- (q$V67)
q$vecuronium<- as.integer(q$V68)
q$Analgesia[q$V69 == '0'] <- 'Control  group'   #renaming

q$Analgesia[q$V69 == '1'] <- 'Class I'
q$Analgesia[q$V69 == '2'] <- 'Class II'
q$Analgesia[q$V69 == '3'] <- 'Class III'
q$cristaloid<- q$V70
q$RBC<- (q$V71)
q$atropin<- (q$V72)
q$efedrin<- (q$V73)

#data visualisation with boxplot
boxplot(QoR40 ~ Epidural, data = q,
    xlab = " ",
    ylab = "QoR-40 ",
    notch = FALSE,
    varwidth = TRUE,
    col = c("orange"," violet"),
    names = c("Control group","Epidural group")
)
boxplot(QoR15 ~ Epidural, data = q,
    xlab = " ",
    ylab = "QoR-15 ",
    notch = FALSE,
    varwidth = TRUE,
    col = c("orange"," violet"),
    names = c("Control group","Epidural group")
)
```

```
boxplot(QoRVAS ~ Epidural, data = q,
    xlab = " ",
    ylab = "QoR-VAS ",
    notch = FALSE,
    varwidth = TRUE,
    col = c("orange"," violet"),
    names = c("Control group","Epidural group")
)

boxplot(Anxiety ~ Epidural, data = q,
    xlab = " ",
    ylab = "VAS anxiety ",
    notch = FALSE,
    varwidth = TRUE,
    col = c("orange"," violet"),
    names = c("Control group","Epidural group")
)

boxplot(PainR ~ Epidural, data = q,
    xlab = " ",
    ylab = "VAS pain at rest ",
    notch = FALSE,
    varwidth = TRUE,
    col = c("orange"," violet"),
    names = c("Control group","Epidural group")
)
boxplot(PainA ~ Epidural, data = q,
    xlab = " ",
    ylab = "VAS pain at activity",
    notch = FALSE,
    varwidth = TRUE,
    col = c("orange"," violet"),
    names = c("Control group","Epidural group")
)

boxplot(Nausea~ Epidural, data = q,
    xlab = " ",
    ylab = "VAS Nausea",
```

```
            notch = FALSE,
            varwidth = TRUE,
            col = c("orange"," violet"),
            names = c("Control group","Epidural group")
        )
```

#Normality analyisis

describe(q[72:92])

the problem with mvn test is that category variable needs to be excluded from analysis or test will #not work. MVN test calculate univariate Shapiro-Wilk test, and descriptives.

```
m1<- mvn(data = q[72:83], mvnTest = c("mardia"), showOutliers = TRUE, univariateTest="SW")
r1 <- mvn(data = q[72:83],univariatePlot = "qqplot")
r2<- mvn(data = q[72:83], univariatePlot = "histogram")

m1<- mvn(data = q[86:89], mvnTest = c("mardia"), showOutliers = TRUE, univariateTest="SW")
r1 <- mvn(data = q[, c(86:89)],univariatePlot = "qqplot")
r2<- mvn(data = q[, c(86:89)], univariatePlot = "histogram")

#-qq plot analysis, shorted for all other variables
ggqqplot(q, x = "QoR40", facet.by = "Epidural")
ggqqplot(q, x = "QoR40
#reliability calculation
QoR40= q[,1:40]
QoR15= q[,41:55]
tab_itemscale(QoR40,show.shapiro = TRUE, show.kurtosis = TRUE,use.viewer= TRUE) #Cronbach's =0.882
psych::alpha(QoR40)
tab_itemscale(QoR15,show.shapiro = TRUE, show.kurtosis = TRUE,use.viewer= TRUE) #Cronbach's =0.727
psych::alpha(QoR15)
```

#correlation analysis

```
mardia<- mvn(data = q[79:85], mvnTest = c("mardia"), showOutliers = TRUE, univariateTest="SW")
chart.Correlation(q[, c(79:85)], method= "spearman", histogram=TRUE, pch=19)
chart.Correlation(q[, c(79:83)], method= "spearman", histogram=TRUE, pch=19)
chart.Correlation(q[, c(83:85)], method= "spearman", histogram=TRUE, pch=19)
chart.Correlation(q[, c(74:79)], method= "spearman", histogram=TRUE, pch=19)
```

descriptive statistics and between group analysis (demographic variables)

```
summary(q)
describe(QoR40 ~ Epidural,IQR=TRUE, data=q)
describe(QoR15 ~ Epidural,IQR=TRUE, data=q)
describe(QoRVAS ~ Epidural,IQR=TRUE, data=q)
describe(Nausea ~ Epidural,IQR=TRUE, data=q)
describe(PainA ~ Epidural,IQR=TRUE, data=q)
describe( Anxiety ~ Epidural,IQR=TRUE, data=q)
describe(q$Age,IQR=TRUE)
describe(q$Timeanest,IQR=TRUE)
describe(q$Timesurgery,IQR=TRUE)
describe(Age ~ Epidural,IQR=TRUE, data=q)
wilcox.test(Age ~ Epidural, data=q,conf.lev=0.95,conf.int=TRUE,exact=F,correct=T)
table(q$ASA,q$Epidural)
wilcox.test(ASA ~ Epidural, data=q,conf.lev=0.95,conf.int=TRUE,exact=F,correct=T)
shapiro.test(q$Timesurgery)
shapiro.test(q$Timeanest)
describe(Timesurgery~ Epidural,IQR=TRUE, data=q)
describe( Timeanest~ Epidural,IQR=TRUE, data=q)
wilcox.test(Timesurgery ~ Epiduraldata=q,conf.lev=0.95,conf.int=TRUE,exact=F,correct=T)
wilcox.test(Timeanest ~ Epidural, data=q,conf.lev=0.95,conf.int=TRUE,exact=F,correct=T)
contingency_table <- table(q$RBC,q$Epidural)
 contingency_table
```

```
fisher.test(contingency_table)

describe( cristaloid ~ Epidural,IQR=TRUE, data=q)

wilcox.test(cristaloid ~ Epidural, data=q,conf.lev=0.95,conf.int=TRUE,exact=F,correct=T)

describe( efedrin ~ Epidural,IQR=TRUE, data=q)

shapiro.test(q$cristaloid)

atropin <- read.delim2("C:/Users/38591/OneDrive/Desktop/atropin.txt", header=FALSE, comment.char="#")  #there was some error with variable atropine, so the column was copy paste in other txt file together with epidural group.

describe( V2 ~ V1,IQR=TRUE, data=atropin)

shapiro.test(atropin$V2)

wilcox.test(V2 ~ V1, data=atropin,conf.lev=0.95,conf.int=TRUE,exact=F,correct=T)

shapiro.test(q$vecuronium)

summary(q$atropin)

shapiro.test(q$vecuronium)

describe(vecuronium ~ Epidural,IQR=TRUE, data=q)

wilcox.test(vecuronium ~ Epidural, data=q,conf.lev=0.95,conf.int=TRUE,exact=F,correct=T)

describe(fentanyl ~ Epidural,IQR=TRUE, data=q)

summary (q$fentanyl)

table(q$fentanyl,q$Epidural)

table(q$Analgesia, q$Epidural)


# Primary outcome

wilcox.test( QoR40 ~ Epidural, data=q,conf.lev=0.95,conf.int=TRUE,exact=F,correct=T)


# Wilcoxon rank sum test with continuity correctin     #data:  QoR40 by Epidural

# W = 403.5, p-value = 0.0001374     # alternative hypothesis: true location shift is not equal to 0

# 95 percent confidence interval:    # -14.000036  -5.000017

#sample estimates:    #  difference in location     # -9.999982


describe(QoR40 ~ Epidural,IQR=TRUE, data=q)

q %>% wilcox_effsize(QoR40 ~ Epidural)
```

Effect size and estimates of the QoR-40 subdimensions

```
wilcox.test( Comfort ~ Epidural, data=q,conf.lev=0.95,conf.int=TRUE,exact=F,correct=T)
q %>% wilcox_effsize(Comfort ~ Epidural)
describe(Comfort ~ Epidural,IQR=TRUE, data=q)

q %>% wilcox_effsize( Emotions~ Epidural)
wilcox.test( Emotions ~ Epidural, data=q,conf.lev=0.95
,conf.int=TRUE,exact=F,correct=T)
describe(Emotions ~ Epidural,IQR=TRUE, data=q)

q %>% wilcox_effsize(PschyologicalSupport ~ Epidural)
wilcox.test( PschyologicalSupport ~ Epidural, data=q,conf.lev=0.95,conf.int=TRUE,exact=F,correct=T)
describe(PschyologicalSupport ~ Epidural,IQR=TRUE, data=q)

q %>% wilcox_effsize( Independence~ Epidural)
wilcox.test( Independence ~ Epidural, data=q,conf.lev=0.95,conf.int=TRUE,exact=F,correct=T)
describe(Independence ~ Epidural,IQR=TRUE, data=q)

q %>% wilcox_effsize( Pain ~ Epidural)
wilcox.test( Pain~ Epidural, data=q,conf.lev=0.95,conf.int=TRUE,exact=F,correct=T)
describe(Pain ~ Epidural,IQR=TRUE, data=q)
```

#Secondary outcomes- multivariate testing

```
q$Epidural=  as.factor(q$Epidural)
independence_test( QoR15 + QoRVAS ~ Epidural, data= q, distribution =
approximate(nresample = 100000))
# maxT 3.9337, p-value = 3e-05
independence_test(PainR +PainA + Anxiety +  Nausea ~ Epidural, data= q, distribution =
approximate(nresample = 100000))
#maxT = 3.5935, p-value = 0.00078
```

#Secondary outcomes, statistic estimates and effect size calculation

```
wilcox.test( Nausea ~ Epidural, data=q,conf.lev=0.95,conf.int=TRUE,exact=F,correct=T)

wilcox.test( QoRVAS~ Epidural, data=q,conf.lev=0.95,conf.int=TRUE,exact=F,correct=T)

wilcox.test( PainR~ Epidural, data=q,conf.lev=0.95,conf.int=TRUE,exact=F,correct=T)

wilcox.test( PainA~ Epidural, data=q,conf.lev=0.95,conf.int=TRUE,exact=F,correct=T)

wilcox.test( QoR15~ Epidural, data=q,conf.lev=0.95,conf.int=TRUE,exact=F,correct=T)

wilcox.test( Anxiety~ Epidural, data=q,conf.lev=0.95,conf.int=TRUE,exact=F,correct=T)

q %>% wilcox_effsize( QoRVAS ~ Epidural)

q %>% wilcox_effsize( PainR ~ Epidural)

q %>% wilcox_effsize( PainA ~ Epidural)

q %>% wilcox_effsize( Nausea ~ Epidural)

q %>% wilcox_effsize( QoR15 ~ Epidural)

q %>% wilcox_effsize( Anxiety ~ Epidural)

describe(PainR ~ Epidural,IQR=TRUE, data=q)

describe(PainA ~ Epidural,IQR=TRUE, data=q)
```

```
#VAS analysis with predetermined cut off points

x= q$PainA

wilcox.test( x, y = NULL, mu = 40,alternative = "less" ,data=q,conf.lev=0.95,conf.int=TRUE,exact=F,correct=T)

x= q$PainR

wilcox.test( x, y = NULL, mu = 30, alternative ="higher", data=q,conf.lev=0.95,conf.int=TRUE,exact=F,correct=T)

q %>% wilcox_effsize( Anxiety ~ Epidural)
```
